# Supplementary material for: Chromothripsis during telomere crisis is independent of NHEJ, and consistent with a replicative origin
Source: Genome Res. 2019 May;29(5):737–49. doi: 10.1101/gr.240705.118 (PMC6499312; doi:10.1101/gr.240705.118)
Supplement: Supplemental Material [file supp_gr.240705.118_Supplemental_file_1.zip › contigs/annotated_contigs/DB91/contig.2.DB91_length_274_mean_cov_1.79562043796.docx]

**DB91_length_274_mean_cov_1.79562043796**

```
A|GAAATTAGCCGGGTGTGGTTGCACATGCTTGTTTTCCCAGCTACTCG|TGATGCTGGAGCA|AGAGGATCGCTTGAGTCCAGGAGTT
  >chr2:7750809-7750856 - E=6e-05                             >chr12:31788407-31788620
CAAGACCAGCATGGGCAACATGACAAAACCCCTCTCAATAAATAAAATAAAAATAAAAGAATACAAATAAATAACTAAATTAGGTAAAC
 - E=3e-111
TGTTGCACTTAATATTTATCTGAACCAGTGGTGTTTATGTCAAGATGGGGTCAGATTCTGGACTAAATCAAACTCTCAGACAAGAAAAT

GGCCTCTTGC
```
